# *Bacillus amyloliquefaciens* Alleviates Clothianidin-Induced Liver Oxidative Stress and Growth Suppression in Goslings

**DOI:** 10.3390/antiox15091092

**Published:** 2026-08-31

**Authors:** Guangquan Li, Kaiqi Weng, Yi Liu, Xianze Wang, Huiying Wang, Daqian He

**Affiliations:** Institute of Animal Husbandry and Veterinary Medicine, Shanghai Academy of Agricultural Science, Shanghai 201106, China; lgqdx123@126.com (G.L.); wengkaiqi@saas.sh.cn (K.W.); liuyi20031194@163.com (Y.L.); wxz13187058389@163.com (X.W.)

**Keywords:** antioxidant defense, *Bacillus amyloliquefaciens*, Clothianidin, goslings, liver injury, metabolomics, oxidative stress, transcriptomics

## Abstract

Clothianidin (CLO), a widely used neonicotinoid insecticide, may pose a health risk to waterfowl through contaminated feed ingredients and agricultural residues. This study investigated whether dietary *Bacillus amyloliquefaciens* (BA) could alleviate CLO-induced growth suppression and liver oxidative injury in goslings. Fourteen-day-old Holdobagy goslings were assigned to five treatments for 21 days: control, 2.5 mg/kg CLO, 2.5 mg/kg CLO plus 60 ppm BA, 10 mg/kg CLO, and 10 mg/kg CLO plus 60 ppm BA. CLO exposure reduced final body weight and average daily gain, increased liver index, elevated serum alkaline phosphatase (AKP), aspartate aminotransferase (AST), and malondialdehyde (MDA) levels, and decreased liver total antioxidant capacity (T-AOC) and superoxide dismutase (SOD) activities (*p* < 0.05). Histological analysis further showed hepatocyte disorganization, vacuolar degeneration, congestion, and inflammatory infiltration, which were partially alleviated by BA supplementation. Serum untargeted metabolomics showed clear separation among treatment groups. CLO exposure altered 130 and 215 differential metabolites at the low and high doses, respectively, whereas BA supplementation regulated 115 and 170 differential metabolites under the corresponding CLO backgrounds (*p* < 0.05). These metabolites were mainly enriched in amino acid metabolism, ATP-binding cassette (ABC) transporters, purine metabolism, riboflavin metabolism, glutathione metabolism, glycerophospholipid metabolism, and fatty acid biosynthesis/degradation pathways. Liver transcriptomic analysis identified 761 and 1283 differentially expressed genes after low- and high-dose CLO exposure, respectively, while BA supplementation altered 338 and 440 genes (*p* < 0.05). Enriched pathways included peroxisome proliferator-activated receptor (PPAR) signaling, fatty acid metabolism, pyruvate metabolism, glycolysis/gluconeogenesis, pentose phosphate pathway, glutathione metabolism, cell adhesion molecules, and adipocytokine signaling. This study adds new evidence on the protective role of BA against CLO-induced toxicity in goslings by combining physiological, histological, metabolomic, and transcriptomic observations. Overall, BA partially mitigated CLO-induced growth inhibition and liver oxidative injury, potentially through coordinated regulation of redox homeostasis, lipid and energy metabolism, and liver metabolic remodeling.

## 1. Introduction

Neonicotinoid insecticides have been widely used in modern agriculture because of their systemic activity, broad insecticidal spectrum, and high affinity for insect nicotinic acetylcholine receptors [1,2]. The CLO, a representative second-generation neonicotinoid, is frequently applied in seed coating and crop protection [3]. Owing to its water solubility, environmental persistence, and systemic distribution in plants, CLO can remain in soil, water, crop residues, and feed-related materials, creating potential exposure routes for non-target animals in agricultural production systems [4].

Birds are vulnerable to neonicotinoid exposure because they may ingest treated seeds, contaminated grains, crop residues, or environmental particles during feeding [5]. A recent meta-analysis showed that neonicotinoid exposure can impair multiple aspects of bird life, including health, behavior, reproduction, and survival [6]. For domestic waterfowl, this concern is particularly relevant because geese may contact pesticide residues through feed ingredients, crop residues, and grazing-associated environments.

The liver is a major organ for xenobiotic metabolism and is therefore a key target of pesticide-induced toxicity [7]. CLO exposure has been associated with oxidative stress, lipid peroxidation, altered antioxidant enzyme activity, and liver injury in experimental animals [8]. When antioxidant defenses are insufficient, oxidative stress may further trigger mitochondrial dysfunction, inflammatory activation, and hepatocellular damage. Thus, liver antioxidant capacity and redox-related metabolic pathways are important endpoints for evaluating CLO toxicity in animals [9].

Probiotic intervention has become a practical strategy for reducing toxicant- or stress-induced oxidative injury [10]. Recent evidence suggests that probiotics can alleviate environmental pollutant-induced toxicity by improving antioxidant defenses, reducing inflammation, supporting intestinal barrier function, and modulating gut microbial metabolism [11]. BA was chosen because it is a spore-forming probiotic with good stability during feed processing and gastrointestinal passage, which makes it suitable for dietary supplementation in poultry production [12]. Compared with many non-spore-forming probiotics such as Lactobacillus and Enterococcus, Bacillus species generally show stronger environmental tolerance and better survival during feed storage and digestion [13]. In addition, BA has been reported to improve intestinal health and reduce inflammatory responses in broilers [14]. Therefore, BA may help alleviate CLO-induced injury through antioxidant, anti-inflammatory, intestinal barrier, and metabolic regulatory effects.

However, current knowledge regarding the effects of CLO on domestic waterfowl, particularly on growth performance and hepatic redox homeostasis in goslings, remains limited. Previous studies have mainly focused on mammals, fish, or model bird species. In contrast, goslings undergo rapid early growth, active metabolism, fast liver development, and relatively high feed intake, which may make their sensitivity to feed-borne pesticide residues different from that of other animals. Therefore, clarifying whether CLO exposure induces growth suppression and hepatic oxidative injury in goslings is important for evaluating the potential risks of neonicotinoid pesticides in waterfowl production systems.

Therefore, in the present study, goslings were used as the experimental model to establish a CLO exposure model, and dietary BA supplementation was applied to evaluate its protective effects. This study mainly examined the effects of CLO exposure and BA intervention on growth performance, hepatic histological injury, oxidative stress indicators, and the expression of antioxidant-related genes/proteins in goslings. We hypothesized that CLO exposure would suppress growth and induce hepatic oxidative damage in goslings, whereas BA supplementation could alleviate CLO-induced adverse effects by enhancing hepatic antioxidant defenses, reducing lipid peroxidation, and improving liver functional status. This study provides experimental evidence for understanding the toxic effects of neonicotinoid pesticides on domestic waterfowl and offers a reference for the application of probiotics in controlling pesticide exposure risks.

## 2. Materials and Methods

### 2.1. Ethics Statement

All animal procedures followed relevant Chinese animal welfare guidelines and were approved by the Experimental Animal Care and Use Committee of the Shanghai Academy of Agricultural Sciences (approval no. SAASPZ0522050). The animal experiment was designed and reported in accordance with the ARRIVE 2.0 guidelines.

### 2.2. Animals, Diets, and Experimental Design

A total of 300 14-day-old male Holdobagy goslings were used in this study. The goslings were provided by Xiangtiange Family Farm, Ma’anshan City, Anhui Province, China.

All goslings were reared under the same management conditions until 14 days of age, with ad libitum access to feed and water. After 14 days of age, the goslings were randomly assigned to five treatment groups: the Con group was fed a basal diet; the C1 group was fed a basal diet supplemented with 2.5 mg/kg CLO; the C1 + BA group was fed a basal diet supplemented with 2.5 mg/kg CLO and 60 ppm BA; the C2 group was fed a basal diet supplemented with 10 mg/kg CLO; and the C2 + BA group was fed a basal diet supplemented with 10 mg/kg CLO and 60 ppm BA. BA was provided as a commercial feed-grade probiotic preparation by Shanghai Jiayi Biotechnology Co., Ltd. (Shanghai, China). The BA preparation contained strain GLD-191 (CGMCC No. 17011) with a viable count of 2 × 10^11^ CFU/g. Each treatment included six replicates with ten goslings per replicate, and the experimental period lasted 21 days. Clothianidin (CAS No. 210880-92-5; purity 98%; catalog No. S25353) was purchased from Shanghai Jiayi Biotechnology Co., Ltd. (Shanghai, China) and used as an analytical-grade compound rather than a commercial insecticide formulation.

The CLO doses were selected to establish a sublethal dietary exposure model rather than to represent acute LD50-based exposure in goslings. Because gosling-specific LD50 data for CLO are unavailable, the dose selection was guided by available avian toxicological data, including reported acute oral and dietary toxicity endpoints in quail and mallard duck. Thus, diets containing 2.5 and 10 mg/kg CLO were used as low and high experimentally induced sublethal exposure levels [15,16,17].

During the experiment, the stocking density was approximately 0.5 m^2^ per gosling, and the house temperature was maintained at around 15 °C. The goslings were exposed to natural light during the day and provided with artificial light at night. Throughout the experiment, all goslings had free access to feed and water. Health status, vaccination, ventilation, and hygiene management were monitored and maintained consistently across all treatment groups. The composition and nutrient levels of the basal diet are shown in Table 1.

### 2.3. Growth Performance

Birds were weighed at the beginning and end of the trial after an 8 h fasting period with free access to water. Feed intake was recorded by replicate. Initial body weight, final body weight, average daily gain (ADG), average daily feed intake (ADFI), and feed/gain ratio (F/G) were calculated.

### 2.4. Sample Collection

At the end of the trial, two goslings with body weights close to the replicate mean were selected from each replicate after an 8 h fasting period, resulting in 12 sampled goslings per group. Blood samples were collected from the wing vein into sterile non-anticoagulant tubes and allowed to clot at room temperature for 30 min. Serum was then separated by centrifugation at 3000× *g* for 10 min at 4 °C and stored at −80 °C until biochemical assays and untargeted metabolomics analysis. Growth performance was analyzed at the replicate level, with six replicates per group. Serum biochemical indices and antioxidant-related indices were analyzed using serum samples from the sampled goslings (*n* = 12 per group). For serum untargeted metabolomics, one gosling from each replicate was selected, resulting in six biological replicates per group (*n* = 6).

After blood collection, the goslings were euthanized by exsanguination before tissue collection. The thymus, spleen, bursa of Fabricius, and liver were immediately collected and weighed. Organ indices were calculated using the sampled goslings (*n* = 12 per group). Liver samples were consistently collected from the same anatomical site of the left liver lobe in each gosling to minimize sampling variation. The samples were rinsed with ice-cold physiological saline and blotted dry. A portion of each liver sample was fixed in 4% paraformaldehyde for histopathology, and representative liver sections were prepared from the sampled goslings. Another portion was snap-frozen in liquid nitrogen and stored at −80 °C for liver antioxidant assays and transcriptomic analysis. Liver antioxidant assays were performed using samples from the sampled goslings (*n* = 12 per group). For liver transcriptomic analysis, one liver sample from each replicate was selected, resulting in six biological replicates per group (*n* = 6).

### 2.5. Serum Biochemical and Antioxidant Analyses

Serum biochemical and antioxidant indicators were determined using commercial assay kits according to the manufacturers’ instructions. Serum biochemical indices, including total protein (TP), albumin (ALB), globulin (GLOB), alanine aminotransferase (ALT), aspartate aminotransferase (AST), alkaline phosphatase (AKP), glucose (GLU), high-density lipoprotein cholesterol (HDL-C), low-density lipoprotein cholesterol (LDL-C), total cholesterol (TC), and triglycerides (TGs), were measured using kits supplied by Shanghai Pinyi Biotechnology Co., Ltd. (Shanghai, China) with an automatic biochemical analyzer (BS-200, Mindray Bio-Medical Electronics Co., Ltd., Shenzhen, China). Serum antioxidant-related indicators, including superoxide dismutase (SOD), catalase (CAT), malondialdehyde (MDA), glutathione peroxidase (GSH-Px), and total antioxidant capacity (T-AOC), were measured using kits purchased from Nanjing Jiancheng Bioengineering Institute (Nanjing, China). The absorbance of antioxidant assays was measured using a microplate reader (Infinite F50, Tecan Group Ltd., Männedorf, Switzerland). All assays were performed strictly following the corresponding kit protocols. The catalog numbers of the assay kits used for each measured parameter are provided in Table S1.

### 2.6. Organ Indices and Liver Antioxidant Assays

The organs were excised, cleaned of adherent tissue, and weighed using an electronic balance (BSM-220A, Shanghai Zhuojing Electronic Technology Co., Ltd., Shanghai, China). Organ index was calculated using the following equation: organ index (g/kg) = organ weight (g)/body weight (kg). Liver homogenates were prepared on ice and used to determine SOD, CAT, GSH-Px, T-AOC, and MDA after normalization to protein concentration.

### 2.7. Liver Histopathology

Fixed liver tissues were dehydrated, embedded in paraffin, sectioned at approximately 4–5 micrometers, stained with hematoxylin and eosin (HE), and observed under a light microscope (BX53, Olympus Corporation, Tokyo, Japan). Histopathological assessment was performed qualitatively based on hepatocyte arrangement, vacuolar degeneration, congestion, and inflammatory cell infiltration.

### 2.8. Serum Untargeted Metabolomics

All serum samples used for metabolomic analysis were stored at −80 °C and analyzed within 2 weeks after collection. Serum samples were subjected to untargeted metabolomic analysis using an ultra-high-performance liquid chromatography system (Vanquish Flex, Thermo Fisher Scientific, Waltham, MA, USA) coupled to a high-resolution mass spectrometer (Orbitrap Exploris 120, Thermo Fisher Scientific, Waltham, MA, USA). Briefly, 50 μL of serum sample was transferred into the 2 mL centrifuge tube, mixed with 200 μL of pre-cooled methanol:acetonitrile (1:1, *v*/*v*), vortexed for 30 s, and then frozen at −20 °C for 30 min. The samples were centrifuged at 12,000 rpm for 10 min at 4 °C, and 200 μL of supernatant was collected and vacuum-dried. The dried extract was reconstituted in 150 μL of 50% methanol containing 5 ppm L-2-chlorophenylalanine, vortexed for 30 s, centrifuged again at 12,000 rpm for 10 min at 4 °C, and filtered through a 0.22 μm membrane before liquid chromatography–mass spectrometry (LC-MS) analysis. Quality control (QC) samples were prepared by pooling 10–20 μL aliquots from each sample filtrate to monitor instrument stability and data reliability.

Chromatographic separation was performed on an ACQUITY ultra-performance liquid chromatography (UPLC) HSS T3 column (100 Å, 1.8 μm, 2.1 mm × 100 mm). The flow rate was 0.4 mL/min, the column temperature was maintained at 40 °C, the autosampler temperature was 8 °C, and the injection volume was 2 μL. The mobile phases consisted of 0.1% formic acid in water as mobile phase A and acetonitrile containing 0.1% formic acid as mobile phase B. The gradient program was as follows: 0–1 min, 5% B; 1–7 min, 5–95% B; 7–8 min, 95% B; 8–8.1 min, 95–5% B; and 8.1–12 min, 5% B.

Mass spectrometric detection was performed using a heated electrospray ionization source in both positive and negative ion modes. The main mass spectrometry (MS) parameters were as follows: spray voltage, 3.5 kV/−3.0 kV; sheath gas, 40 arb; auxiliary gas, 10 arb; capillary temperature, 320 °C; auxiliary gas temperature, 300 °C; MS1 resolution, 60,000; scan range, *m*/*z* 70–1000; automatic gain control (AGC) target, standard; maximum injection time, 100 ms; top 4 ions selected for tandem mass spectrometry (MS/MS) fragmentation; dynamic exclusion time, 4 s; MS2 resolution, 15,000; higher-energy collisional dissociation (HCD) collision energy, 30%; MS2 AGC target, standard; and MS2 maximum injection time, auto.

Raw LC-MS data were processed using MS-DIAL software (version 4.9.221218) for peak extraction, alignment, filtering, normalization, and metabolite identification. Compounds missing in more than 50% of samples within each group were filtered out, and undetected peaks were imputed and normalized. Metabolite annotation was performed using the PerSonalbio Next-Generation Metabolomics Database, which includes an in-house standard library, mzCloud, LIPID MAPS, Human Metabolome Database (HMDB), MassBank of North America (MoNA), NIST_2020_MSMS, and an AI-predicted MS/MS spectral library. The main search parameters included MS1 tolerance for identification of 0.01, MS2 tolerance for identification of 0.05, smoothing level of 3, minimum peak height of 10,000, minimum peak width of 5, mass slice width of 0.05, and identification score cut-off of 70.

The processed metabolite matrix was analyzed using Personalbio GenesCloud (www.genescloud.cn). Peaks with a relative standard deviation greater than 30% in QC samples were excluded. Principal component analysis (PCA) was performed using ropls (version 4.3.3) to evaluate sample distribution and analytical stability. Orthogonal partial least squares–discriminant analysis (OPLS-DA) was used to assess metabolic separation among treatment groups. Differential metabolites were screened based on Variable Importance in Projection (VIP) > 1.0 and *p* < 0.05. Identified differential metabolites were further subjected to Kyoto Encyclopedia of Genes and Genomes (KEGG) pathway enrichment analysis using clusterProfiler (version 4.6.0).

### 2.9. Liver Transcriptomics

Total RNA was extracted from liver tissue samples using a standard RNA extraction protocol. RNA concentration and purity were assessed using spectrophotometry (Thermo Fisher Scientific, Waltham, MA, USA), and RNA integrity was evaluated before library construction. Only RNA samples with acceptable purity and integrity were used for subsequent sequencing.

Sequencing libraries were constructed according to the manufacturer’s instructions and sequenced on the (Vazyme Biotech Co., Ltd., Nanjing, China) using a paired-end sequencing strategy. The average sequencing depth was approximately 30 million clean reads per sample.

Raw sequencing reads were filtered to remove adapter sequences, low-quality reads, and reads with excessive unknown bases. The resulting clean reads were aligned to the goose reference genome using HISAT2 v2.2.1. Gene expression levels were quantified using featureCounts in the Subread package v2.0.6 and normalized to generate the expression matrix for each sample. Differentially expressed genes were identified using DESeq2 v1.42.0. Genes with an adjusted *p*-value < 0.05 and an absolute log2 fold change > 1 were considered significantly differentially expressed. Functional enrichment analysis of DEGs was performed using the KEGG database.

### 2.10. Data Analysis

The experimental data were analyzed using SPSS 26.0 software (IBM Corp., Armonk, NY, USA). Data normality was assessed using the Shapiro–Wilk test, and homogeneity of variance was evaluated using Levene’s test before statistical analysis. When the assumptions of normality and homogeneity of variance were satisfied, differences among treatment groups were analyzed using one-way analysis of variance (ANOVA), followed by Duncan’s multiple range test when significant treatment effects were detected. When these assumptions were not satisfied, data were transformed before ANOVA; if the assumptions remained unmet after transformation, a non-parametric Kruskal–Wallis test was used. Data are presented as treatment means with the pooled standard error of the mean (SEM) shown in a separate column. A value of *p* < 0.05 was considered statistically significant. Different lowercase letters within the same row indicate significant differences among groups (*p* < 0.05).

For omics datasets, multiple testing correction was applied where appropriate. In transcriptomic analysis, *p*-values were adjusted using the Benjamini–Hochberg false discovery rate (FDR) method, and genes with an adjusted *p*-value < 0.05 and an absolute log2 fold change > 1 were considered differentially expressed. For untargeted metabolomics, differential metabolites were screened based on VIP > 1.0 and *p* < 0.05, and pathway enrichment analysis was adjusted for multiple testing using the Benjamini–Hochberg FDR method.

## 3. Results

### 3.1. Growth Performance

As shown in Table 2, there was no significant difference in initial body weight among the five treatment groups. Dietary treatment significantly affected final body weight and ADG (*p* < 0.05). Compared with the Con group, the C1, C1 + BA, C2, and C2 + BA groups showed decreasing trends in final body weight and ADG, with the lowest values observed in the C2 group. No significant difference was observed in F/G among the groups (*p* > 0.05). In addition, dietary treatment significantly affected the ADFI (*p* < 0.05), with the lowest value observed in the C2 group.

### 3.2. Organ Indices

As shown in Table 3, different dietary treatments significantly affected the liver index of goslings (*p* < 0.05). The liver indices in the C2 and C2 + BA groups were significantly higher than those in the Con, C1, and C1 + BA groups (*p* < 0.05), indicating that high-dose CLO treatment may increase the relative liver weight of goslings. In contrast, no significant differences were observed in the spleen index, thymus index, or bursa of Fabricius index among the groups (*p* > 0.05), suggesting that the different treatments had no significant effect on the indices of the major immune organs.

### 3.3. Serum Biochemical and Antioxidant Indices

**Figure 1:**
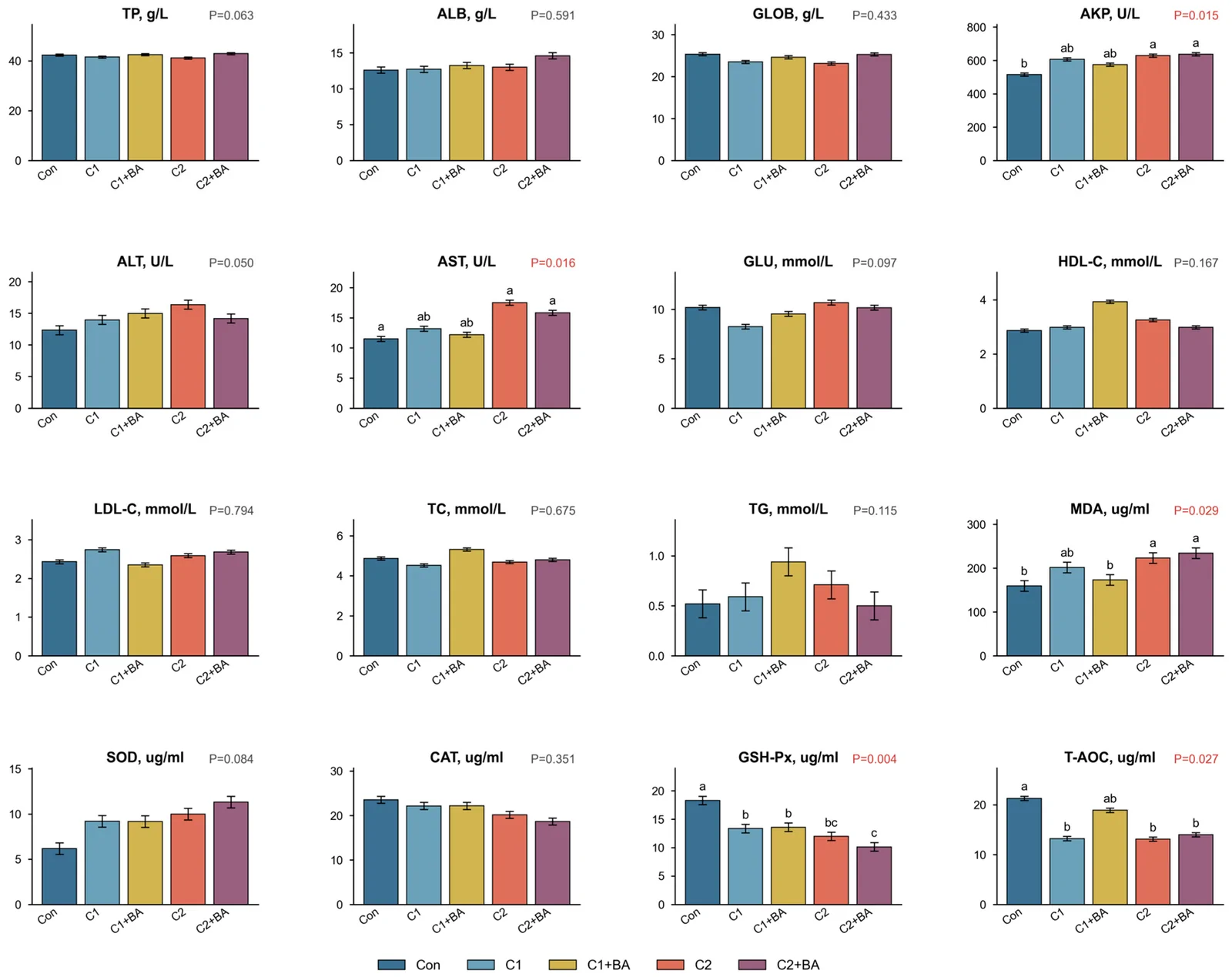
Effects of BA supplementation on serum biochemical and antioxidant indices in CLO-exposed goslings (*n* = 12 goslings per group). Con, control group; C1, 2.5 mg/kg CLO group; C1 + BA, 2.5 mg/kg CLO plus 60 ppm BA group; C2, 10 mg/kg CLO group; C2 + BA, 10 mg/kg CLO plus 60 ppm BA group. Data are presented as means ± SEM. TP, total protein; ALB, albumin; GLOB, globulin; ALT, alanine aminotransferase; AST, aspartate aminotransferase; AKP, alkaline phosphatase; GLU, glucose; HDL-C, high-density lipoprotein cholesterol; LDL-C, low-density lipoprotein cholesterol; TC, total cholesterol; TG, triglycerides; SOD, superoxide dismutase; CAT, catalase. Colored text in the panels indicates the corresponding *p*-values of pairwise comparisons. Different lowercase letters above bars indicate significant differences among groups (*p* < 0.05).

As shown in Figure 1, dietary treatments significantly affected several serum biochemical and oxidative stress-related indicators. No significant differences were observed among the groups in TP, ALB, GLOB, ALT, GLU, HDL-C, LDL-C, TC, TG, SOD, or CAT levels (*p* > 0.05), indicating that the different treatments had limited effects on most basic serum biochemical parameters. In contrast, AKP and AST levels differed significantly among groups (*p* < 0.05). AKP levels were relatively higher in the C2 and C2 + BA groups, while the highest AST level was observed in the C2 group.

For oxidative stress-related indicators, MDA levels differed significantly among groups (*p* < 0.05), with higher values observed in the C2 and C2 + BA groups, suggesting increased lipid peroxidation. GSH-Px and T-AOC levels were also significantly affected by dietary treatment. Compared with the Con group, CLO treatment decreased GSH-Px and T-AOC levels, with the lowest GSH-Px level observed in the C2 + BA group.

### 3.4. Liver Antioxidant Level

**Figure 2:**
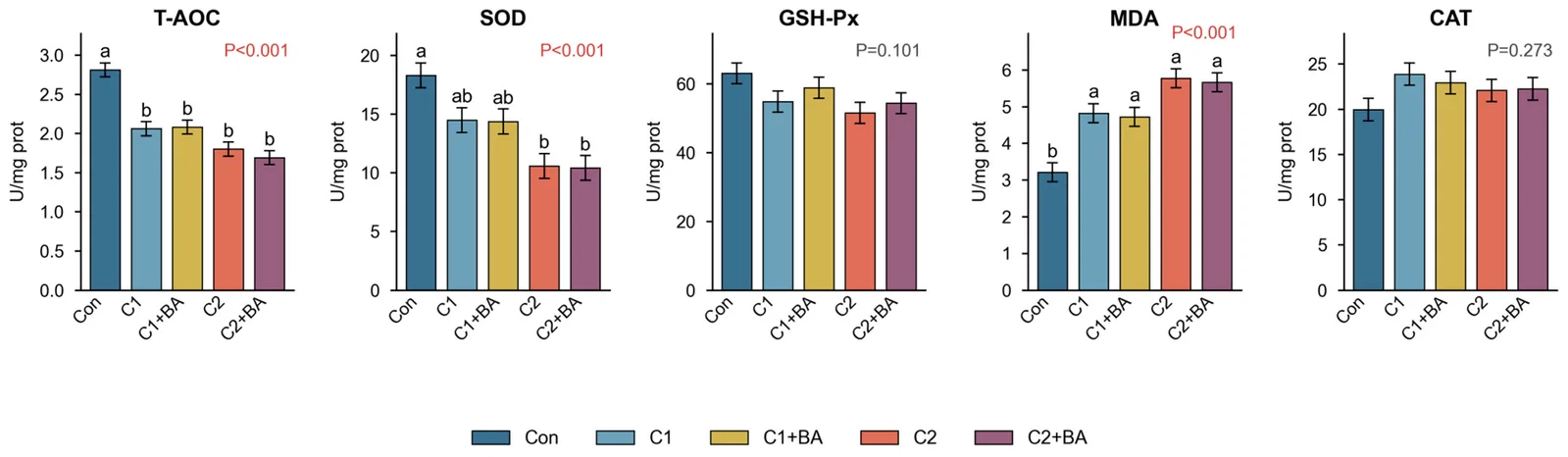
Effects of BA supplementation on liver antioxidant indices in CLO-exposed goslings. Con, control group (*n* = 12 goslings per group); C1, 2.5 mg/kg CLO group; C1 + BA, 2.5 mg/kg CLO plus 60 ppm BA group; C2, 10 mg/kg CLO group; C2 + BA, 10 mg/kg CLO plus 60 ppm BA group. Data are presented as means ± SEM. T-AOC, total antioxidant capacity; SOD, superoxide dismutase; MDA, malondialdehyde; GSH-Px, glutathione peroxidase; CAT, catalase. Colored text in the panels indicates the corresponding *p*-values of pairwise comparisons. Different lowercase letters above bars indicate significant differences among groups (*p* < 0.05).

As shown in Figure 2, dietary treatment significantly affected liver T-AOC, SOD, and MDA levels (*p* < 0.05), whereas no significant effects were observed on GSH-Px or CAT activities (*p* > 0.05). Compared with the Con group, hepatic T-AOC levels were significantly decreased in the C1, C1 + BA, C2, and C2 + BA groups (*p* < 0.05). SOD activity also showed a decreasing trend, with significantly lower levels observed in the C2 and C2 + BA groups than in the Con group (*p* < 0.05). In contrast, hepatic MDA levels were significantly increased in the CLO-treated and CLO + BA-treated groups (*p* < 0.05), with relatively higher values observed in the C2 and C2 + BA groups. No significant differences in GSH-Px or CAT activities were observed among the groups.

### 3.5. Liver Histopathology

**Figure 3:**
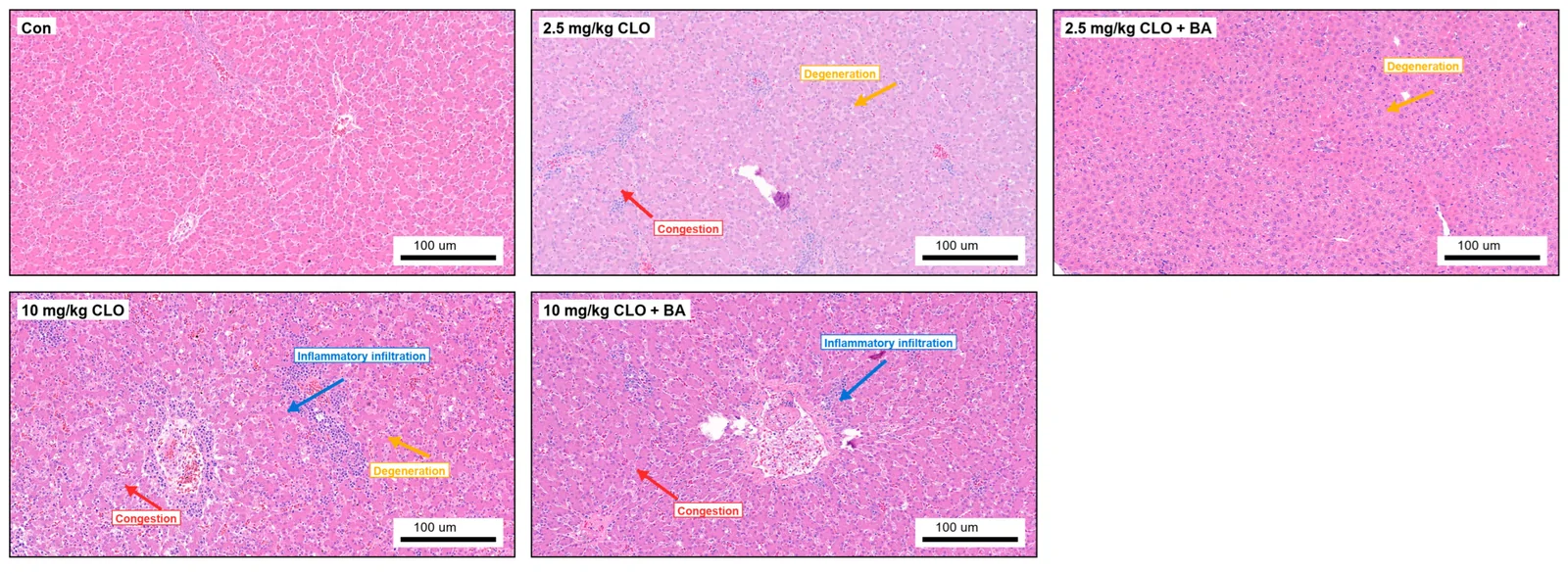
Representative HE-stained liver sections of goslings from different treatment groups (*n* = 12 goslings per group). All images were captured at 40.0× magnification. Arrows indicate hepatocyte degeneration, congestion, and inflammatory infiltration. Scale bar = 100 μm. HE, hematoxylin and eosin.

As shown in Figure 3, the HE staining showed that the liver tissue structure in the Con group was relatively intact, with regularly arranged hepatocytes. In the C1 group, mild pathological alterations were observed, including disorganized hepatocyte arrangement, localized vacuolar degeneration, and slight inflammatory cell infiltration. Compared with the C1 group, the C1 + BA group showed relatively improved liver morphology, with reduced inflammatory cell infiltration. More obvious hepatic injury was observed in the C2 group, characterized by marked inflammatory cell infiltration, local congestion, and hepatocyte degeneration. In contrast, the C2 + BA group showed less severe pathological changes than the C2 group.

### 3.6. Serum Metabolomics

**Figure 4:**
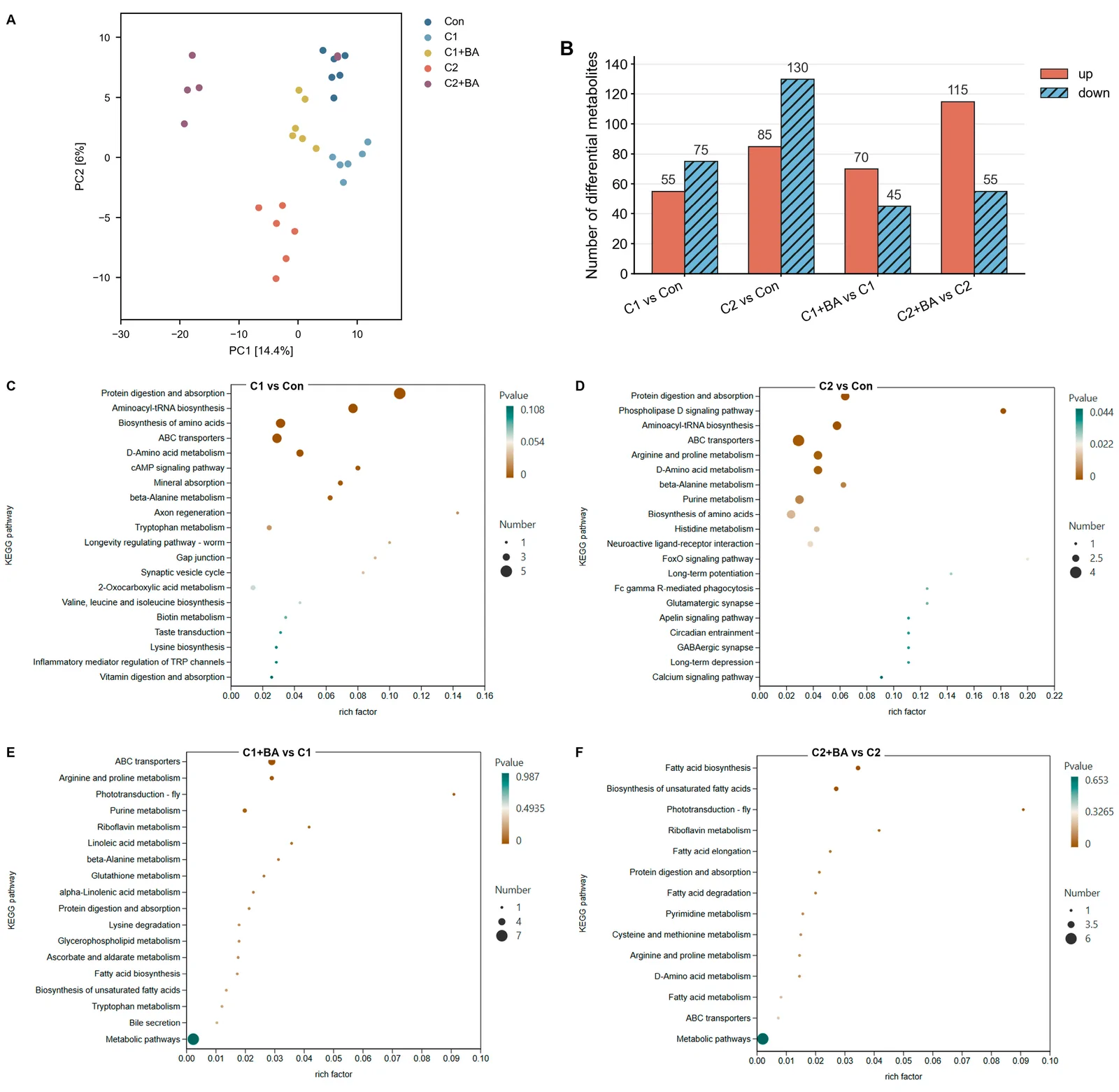
Serum untargeted metabolomic analysis of goslings exposed to CLO with or without BA supplementation (*n* = 6 biological replicates per group). (**A**) PCA score plot; (**B**) Number of differential metabolites; (**C**–**F**) KEGG enrichment plots for C1 vs. Con, C2 vs. Con, C1 + BA vs. C1 and C2 + BA vs. C2, respectively.

As shown in Figure 4A, the OPLS-DA score plot revealed a clear separation of serum metabolic profiles among the five treatment groups. Samples within each group showed good clustering, whereas clear differences were observed among groups.

The statistical analysis of differential metabolites is shown in Figure 4B. A total of 130 differential metabolites were identified in C1 vs. Con, including 55 up-regulated and 75 down-regulated metabolites. In C2 vs. Con, 215 differential metabolites were detected, including 85 up-regulated and 130 down-regulated metabolites. In C1 + BA vs. C1, 115 differential metabolites were identified, with 70 up-regulated and 45 down-regulated metabolites. In C2 + BA vs. C2, 170 differential metabolites were detected, including 115 up-regulated and 55 down-regulated metabolites.

KEGG enrichment analysis showed that the differential metabolites in C1 vs. Con were mainly enriched in protein digestion and absorption, aminoacyl-tRNA biosynthesis, biosynthesis of amino acids, ABC transporters, D-amino acid metabolism, and beta-alanine metabolism (Figure 4C). The differential metabolites in C2 vs. Con were mainly enriched in protein digestion and absorption, phospholipase D signaling pathway, aminoacyl-tRNA biosynthesis, ABC transporters, arginine and proline metabolism, purine metabolism, and biosynthesis of amino acids (Figure 4D). In C1 + BA vs. C1, the differential metabolites were mainly enriched in ABC transporters, arginine and proline metabolism, purine metabolism, riboflavin metabolism, linoleic acid metabolism, glutathione metabolism, and glycerophospholipid metabolism (Figure 4E). In C2 + BA vs. C2, the differential metabolites were mainly enriched in fatty acid biosynthesis, biosynthesis of unsaturated fatty acids, riboflavin metabolism, fatty acid elongation, fatty acid degradation, pyrimidine metabolism, cysteine and methionine metabolism, and ABC transporters (Figure 4F).

### 3.7. Liver Transcriptomics

**Figure 5:**
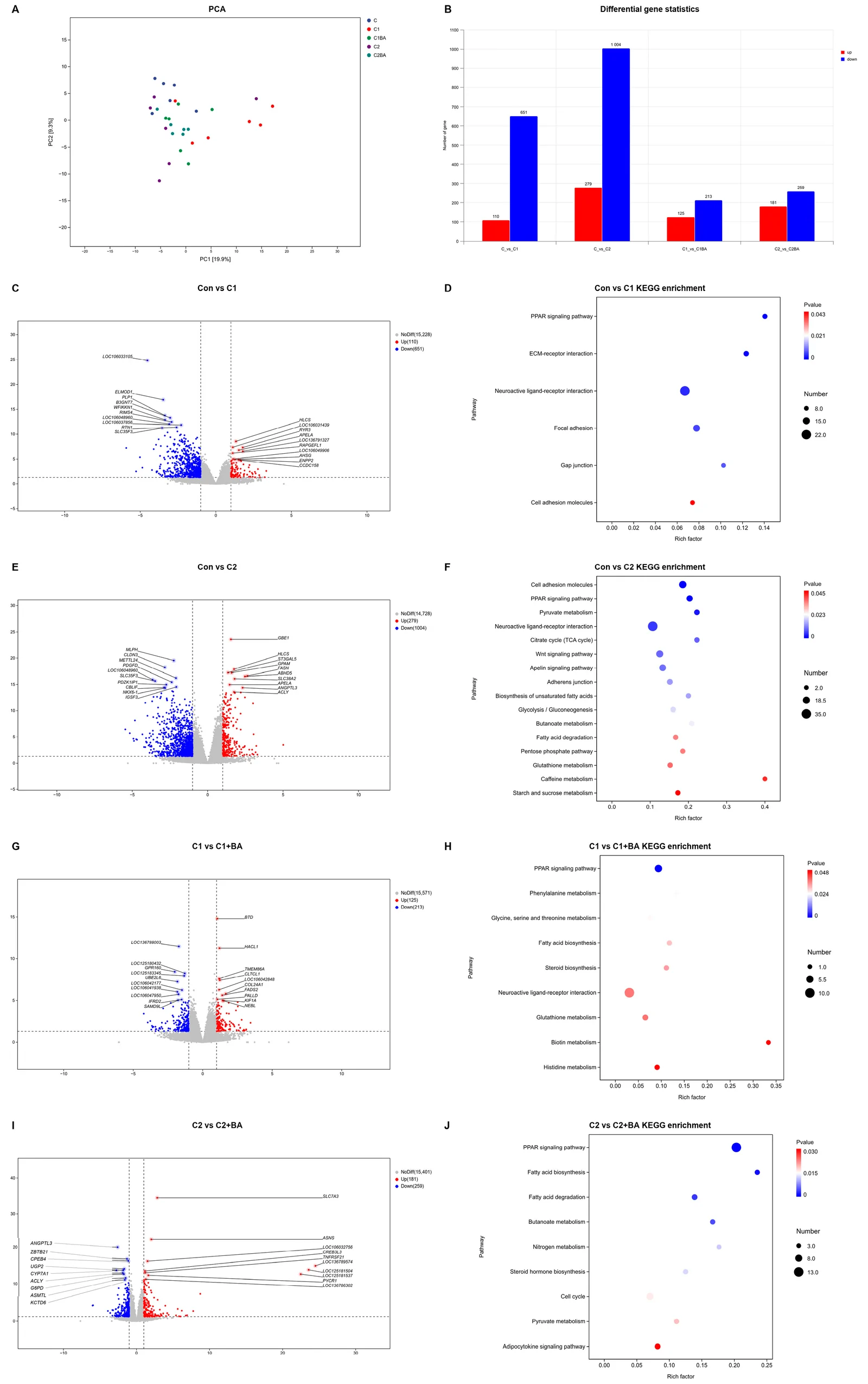
Liver transcriptomic analysis of goslings exposed to CLO with or without BA supplementation (*n* = 6 biological replicates per group). (**A**) PCA plot; (**B**) Statistics of differentially expressed genes; (**C**,**E**,**G**,**I**) Volcano plots for Con vs. C1, Con vs. C2, C1 vs. C1 + BA and C2 vs. C2 + BA, respectively; (**D**,**F**,**H**,**J**) KEGG enrichment analyses for Con vs. C1, Con vs. C2, C1 vs. C1 + BA and C2 vs. C2 + BA, respectively.

As shown in Figure 5A, PCA revealed a separation trend in liver transcriptomic profiles among the five treatment groups. The Con samples were mainly distributed in the upper-left region, while the C1 samples were primarily located on the right side. The C1 + BA and C2 + BA samples were mainly clustered in the central region, whereas the C2 samples showed a relatively dispersed distribution. Differences along PC1 and PC2 among treatment groups indicated that hepatic transcriptomic profiles were altered by CLO exposure and BA supplementation.

The statistics of differentially expressed genes (DEGs) are shown in Figure 5B. A total of 761 DEGs were identified in Con vs. C1, including 110 up-regulated and 651 down-regulated genes. In Con vs. C2, 1283 DEGs were detected, including 279 up-regulated and 1004 down-regulated genes. In C1 vs. C1 + BA, 338 DEGs were identified, including 125 up-regulated and 213 down-regulated genes. In C2 vs. C2 + BA, 440 DEGs were detected, including 181 up-regulated and 259 down-regulated genes. Compared with Con vs. C1, Con vs. C2 showed a larger number of DEGs.

Volcano plots showed different numbers of significantly up-regulated and down-regulated genes across the pairwise comparisons. In Con vs. C1, the number of down-regulated genes was greater than that of up-regulated genes, and representative DEGs included LOC106033105, HLCS, RYR3, APELA, and AHSG (Figure 5C). In Con vs. C2, down-regulated genes were also predominant, with representative DEGs including GBE1, FASN, ANGPTL3, ACLY, MLPH, CLDN3, and METTL24 (Figure 5E). In C1 vs. C1 + BA, the number of DEGs was relatively smaller, and representative DEGs included BTD, HACL1, TMEM86A, FADS2, PALLD, and UBE2L6 (Figure 5G). In C2 vs. C2 + BA, representative up-regulated genes included SLC7A3, ASNS, TNFRSF21, PYCR1, and LOC136786302, whereas representative down-regulated genes included ANGPTL3, ZBTB21, CPEB4, UGP2, CYP7A1, ACLY, G6PD, ASMTL, and KCTD6 (Figure 5I).

KEGG enrichment analysis showed that DEGs in Con vs. C1 were mainly enriched in the PPAR signaling pathway, ECM–receptor interaction, neuroactive ligand–receptor interaction, focal adhesion, gap junction, and cell adhesion molecules (Figure 5D). DEGs in Con vs. C2 were mainly enriched in cell adhesion molecules, PPAR signaling pathway, pyruvate metabolism, neuroactive ligand–receptor interaction, citrate cycle, Wnt signaling pathway, apelin signaling pathway, adherens junction, biosynthesis of unsaturated fatty acids, glycolysis/gluconeogenesis, fatty acid degradation, pentose phosphate pathway, and glutathione metabolism (Figure 5F). In C1 vs. C1 + BA, DEGs were mainly enriched in the PPAR signaling pathway, phenylalanine metabolism, glycine, serine and threonine metabolism, fatty acid biosynthesis, steroid biosynthesis, neuroactive ligand–receptor interaction, glutathione metabolism, biotin metabolism, and histidine metabolism (Figure 5H). In C2 vs. C2 + BA, DEGs were mainly enriched in the PPAR signaling pathway, fatty acid biosynthesis, fatty acid degradation, butanoate metabolism, nitrogen metabolism, steroid hormone biosynthesis, cell cycle, pyruvate metabolism, and adipocytokine signaling pathway (Figure 5J). Overall, the DEGs were mainly associated with lipid metabolism, energy metabolism, cell adhesion, PPAR signaling, and glutathione metabolism-related pathways.

## 4. Discussion

This study showed that CLO exposure suppressed gosling growth and caused evident hepatic injury. Compared with the control group, CLO treatment reduced final body weight and average daily gain, with a more pronounced decrease observed in the high-dose CLO group. Although neonicotinoid insecticides primarily target insect nicotinic acetylcholine receptors, their residues can enter birds through treated seeds, feed ingredients, and agricultural environments and may adversely affect avian behavior, physiological status, reproduction, and survival [20,21]. Goslings are in a rapid growth stage, during which nutrient metabolism is active and hepatic detoxification capacity is under high demand [22]. Therefore, they may be sensitive to feed-borne pesticide residues. The reduced growth performance observed in this study indicates that CLO exposure may pose a potential risk to early growth in goslings.

The liver is the major organ responsible for xenobiotic metabolism and detoxification and is also an important target of pesticide-induced toxicity [23,24]. In the present study, high-dose CLO increased the liver index and altered serum AKP and AST levels. Increased AST generally indicates impaired hepatocyte membrane integrity, whereas changes in AKP are associated with hepatobiliary metabolism and liver injury [25]. Histological observations further showed that CLO exposure caused disordered hepatocyte arrangement, vacuolar degeneration, local congestion, and inflammatory cell infiltration. These pathological changes were consistent with the serum biochemical alterations, suggesting that CLO exposure increased hepatic metabolic burden and caused structural liver injury in goslings.

Oxidative stress may be an important mechanism underlying CLO-induced liver injury [26]. CLO treatment increased serum and hepatic MDA levels, while reducing hepatic T-AOC and SOD activities. MDA is a major product of lipid peroxidation, and its elevation indicates oxidative damage to membrane lipids [27,28]. In contrast, decreased SOD activity and T-AOC suggest a weakened capacity to scavenge reactive oxygen species and maintain redox homeostasis [29]. When reactive oxygen species production exceeds antioxidant defense capacity, cellular membranes, mitochondria, and proteins may be damaged, leading to inflammatory responses and hepatocyte degeneration. Therefore, CLO-induced redox imbalance may be an important link among liver dysfunction, histological injury, and growth suppression.

As a spore-forming probiotic, BA has been widely reported to improve growth performance and antioxidant capacity in poultry. Wang et al. found that supplementation with BA CECT 5940 in a corn–wheat–soybean meal diet increased the activities of antioxidant enzymes, including SOD and GSH-Px, in the serum and liver of broilers at 42 days of age, reduced serum MDA content, and improved growth performance, immune status, and digestive enzyme activity [30]. The study also reported that BA LFB112 increased body weight and average daily gain, decreased the feed conversion ratio, and improved serum biochemical parameters such as total protein, glucose, cholesterol, triglycerides, urea, and creatinine in broilers, suggesting that BA may promote growth by improving nutrient metabolism and overall health status [31]. In addition, BA SC06 increased hepatic T-AOC and T-SOD activities and improved intestinal digestive enzyme activity and immune-related indicators in broilers, indicating a positive effect of BA on hepatic antioxidant status [32].

The protective effects of BA have also been further confirmed in stress or toxic injury models. Li et al. found that BA supplementation alleviated immunological stress-induced growth suppression and improved intestinal morphology and inflammatory responses in lipopolysaccharide-challenged broilers [14]. Recent studies have also shown that BA can alleviate lipopolysaccharide-induced oxidative stress in broilers by increasing serum T-AOC, CAT, and GSH-Px levels and reducing MDA content, while improving intestinal morphology and gut microbial composition [33]. In terms of toxicant-induced liver injury, Li et al. found that BA B10 alleviated aflatoxin B1-induced hepatic oxidative damage and apoptosis in mice, as reflected by improvements in hepatic antioxidant indices, pathological changes, and the expression of apoptosis-related genes and proteins [34]. Another study further reported that BA alleviated aflatoxin B1-induced hepatocyte pyroptosis and oxidative injury by activating the Nrf2/HO-1 signaling pathway [35]. Collectively, these studies indicate that BA can improve antioxidant status and growth performance under normal feeding conditions and can also reduce oxidative damage and liver injury under toxicant or inflammatory challenge conditions.

BA supplementation alleviated, to some extent, the adverse effects induced by CLO. Compared with CLO treatment alone, BA supplementation reduced the severity of hepatic pathological lesions and improved some oxidative stress-related indicators. However, the protective effect of BA was not complete. In this study, some growth and oxidative stress indicators in the BA-supplemented groups did not fully return to control levels, especially under high-dose CLO exposure. This suggests that the beneficial effect of BA may be influenced by CLO dose, exposure duration, and the severity of tissue damage.

Serum metabolomics provided further evidence that CLO exposure induced systemic metabolic disturbance in goslings. After CLO exposure, differential metabolites were mainly enriched in amino acid metabolism, protein digestion and absorption, aminoacyl-tRNA biosynthesis, ABC transporters, purine metabolism, and glycerophospholipid metabolism. These pathways are closely related to nutrient utilization, protein synthesis, xenobiotic transport, energy metabolism, and membrane stability. Under CLO challenge, animals may require more amino acids and energy for detoxification, antioxidant defense, and tissue repair, which may interfere with normal growth [36]. Meanwhile, changes in glycerophospholipid metabolism were consistent with the increased MDA levels, suggesting that membrane lipid peroxidation may contribute to CLO-induced hepatocyte injury [8]. After BA intervention, differential metabolites were mainly involved in glutathione metabolism, riboflavin metabolism, fatty acid metabolism, and cysteine and methionine metabolism. These pathways are closely associated with antioxidant defense [37]. Glutathione is an important intracellular antioxidant involved in peroxide clearance and xenobiotic detoxification. Cysteine and methionine provide substrates for glutathione synthesis, while riboflavin participates in multiple redox reactions and energy metabolism [38]. Therefore, BA may enhance the capacity to eliminate oxidative products by improving the supply of antioxidant-related metabolites, thereby reducing CLO-induced hepatic oxidative damage.

The liver transcriptomic results were generally consistent with the metabolomic findings. CLO exposure significantly altered hepatic gene expression profiles, and the differentially expressed genes were mainly enriched in PPAR signaling, fatty acid metabolism, pyruvate metabolism, glycolysis/gluconeogenesis, the pentose phosphate pathway, and glutathione metabolism. The PPAR signaling pathway is involved in hepatic lipid metabolism and energy homeostasis [39]. The pentose phosphate pathway provides reducing power for antioxidant reactions, whereas glutathione metabolism is directly related to cellular antioxidant capacity [40]. Thus, the concordant changes in metabolomic and transcriptomic profiles suggest that CLO-induced hepatic injury is closely associated with impaired redox balance, altered lipid metabolism, and disturbed energy homeostasis. In this context, BA supplementation may help restore hepatic metabolic adaptation under CLO exposure by modulating antioxidant-related metabolism and lipid utilization. However, these omics-based findings should be interpreted as pathway-level evidence, and targeted validation of key genes, proteins, and metabolites is still needed.

## 5. Conclusions

Dietary exposure to 2.5 and 10 mg/kg CLO impaired growth performance and induced hepatic oxidative injury in goslings, with stronger effects observed at 10 mg/kg CLO. CLO significantly reduced final body weight and ADG, increased liver index, elevated serum AST, AKP, and MDA levels, and decreased hepatic antioxidant capacity, suggesting that growth suppression may be related to oxidative stress-induced metabolic dysfunction and hepatic impairment. Supplementation with 60 ppm BA showed potential to partially alleviate CLO-induced growth suppression and liver oxidative injury, but it did not completely reverse CLO-induced toxicity. Serum metabolomic and liver transcriptomic analyses indicated that glutathione metabolism, lipid and energy metabolism, PPAR signaling, and fatty acid metabolism may be involved in the observed responses to CLO exposure and BA supplementation. Because intestinal microbiota, intestinal morphology, digestive enzyme activity, and barrier integrity were not evaluated, the involvement of gut-related probiotic mechanisms remains a hypothesis that requires further investigation. These findings suggest that BA may be a promising feed additive candidate for reducing the toxicological impact of environmental neonicotinoid exposure in gosling production.

## Figures and Tables

**Table 1:** Composition and nutrient level of experiment diets (air-dry basis).

Items	Content (%)
Ingredients	
Corn	67.92
Soybean meal (43% crude protein)	24.90
Soybean oil	2.00
Lys (98%)	0.09
Met (98%)	0.09
Vitamin–mineral premix ^1^	5.00
Total	100.00
Nutrient level ^2^	
CP	16.00
ME (MJ/kg) ^3^	12.40
CF	2.56
Ca	0.79
P	0.51
Lys	0.90
Met	0.45
Thr	0.63
Cys	0.21

^1^ One kilogram of the premix contained the following: NaCl 4 g, Fe 100 mg, Cu 8 mg, Mn 120 mg, Zn 100 mg, Se 0.4 mg, Co 1.0 mg, I 0.4 mg, VA 8330 IU, VB_1_ 2.0 mg, VB_2_ 0.8 mg, VB_6_ 1.2 mg, VB_12_ 0.03 mg, VD_3_ 1440 IU, VE 30 IU, biotin 0.2 mg, folic acid 2.0 mg, calcium pantothenic acid 20 mg, niacin acid 40 mg. ^2^ CP (crude protein), ME (metabolizable energy), CF (crude fiber). ^3^ Values of nutrient levels were calculated based on the feed ingredient composition according to NRC (1994) and the Chinese feeding standard of meat-producing geese (NY/T 4641-2025) [18,19].

**Table 2:** Effects of BA on growth performance in CLO-exposed goslings (*n* = 6 replicate pens per group).

Items	Con	C1	C1 + BA	C2	C2 + BA	SEM	*p*-Value
Initial BW, g	812.58	812.03	808.23	816.45	796.45	11.51	0.777
Final BW, g	2485.10 ^a^	2294.50 ^ab^	2306.45 ^ab^	2137.62 ^b^	2199.48 ^b^	37.64	<0.001
ADG, g/d	79.64 ^a^	70.59 ^b^	71.34 ^b^	62.91 ^c^	66.81 ^ab^	1.91	<0.001
ADFI, g/d	310.71 ^a^	292.56 ^ab^	301.96 ^ab^	260.24 ^b^	269.11 ^b^	5.21	0.002
F/G	4.53	4.51	4.42	4.37	4.25	0.06	0.682

Note: Different lowercase letters (a, b, c) within a row indicate significant differences (*p* < 0.05).

**Table 3:** Effects of BA on organ indices in CLO-exposed goslings (*n* = 12 goslings per group).

Items	Con	C1	C1 + BA	C2	C2 + BA	SEM	*p*-Value
Liver index	16.61 ^b^	17.15 ^b^	17.85 ^b^	20.52 ^a^	19.98 ^a^	1.12	0.048
Spleen index	1.39	1.01	1.12	0.99	0.95	0.06	0.473
Thymus index	1.43	1.19	1.19	1.42	1.56	0.13	0.533
Bursa index	0.74	0.50	0.53	0.63	0.60	0.05	0.484

Note: Different lowercase letters (a, b) within a row indicate significant differences (*p* < 0.05).

## Data Availability

The sequencing data supporting our findings have been deposited in NGDC with accession number PRJCA066897. Further inquiries can be directed to the corresponding authors.

## Supplementary Materials

The following supporting information can be downloaded at: https://www.mdpi.com/article/10.3390/antiox15091092/s1, Table S1: Catalog numbers and detection limits or detection ranges of assay kits used for serum biochemical and antioxidant analyses.
