# Supplementary material for: Bacillus amyloliquefaciens Alleviates Clothianidin-Induced Liver Oxidative Stress and Growth Suppression in Goslings
Source: Antioxidants (Basel). 2026 Aug 31;15(9):1092. doi: 10.3390/antiox15091092 (PMC13603244; doi:10.3390/antiox15091092)
Supplement: Supplementary file 1 [file antioxidants-15-01092-s001.zip › antioxidants-4413275-supplementary.pdf]

Table S1. Catalog numbers and detection limits or detection ranges of assay kits used for serum biochemical and antioxidant analyses.

Parameter	Assay kit	Catalog No.	Manufacturer	Detection range and sensitivity
TP	Total protein assay kit		Shanghai Pinyi Biotechnology Co., Ltd., Shanghai, China	
ALB	Albumin assay kit		Shanghai Pinyi Biotechnology Co., Ltd., Shanghai, China	
GLOB	Globulin assay kit		Shanghai Pinyi Biotechnology Co., Ltd., Shanghai, China	
ALT	Alanine aminotransferase assay kit		Shanghai Pinyi Biotechnology Co., Ltd., Shanghai, China	
AST	Aspartate aminotransferase assay kit		Shanghai Pinyi Biotechnology Co., Ltd., Shanghai, China	
AKP	Alkaline phosphatase assay kit		Shanghai Pinyi Biotechnology Co., Ltd., Shanghai, China	
GLU	Glucose assay kit		Shanghai Pinyi Biotechnology Co., Ltd., Shanghai, China	
HDL-C	High-density lipoprotein cholesterol assay kit		Shanghai Pinyi Biotechnology Co., Ltd., Shanghai, China	
LDL-C	Low-density lipoprotein cholesterol assay kit		Shanghai Pinyi Biotechnology Co., Ltd., Shanghai, China	
TC	Total cholesterol assay kit		Shanghai Pinyi Biotechnology Co., Ltd., Shanghai, China	
TG	Triglyceride assay kit		Shanghai Pinyi Biotechnology Co., Ltd., Shanghai, China	
SOD	Total superoxide dismutase assay kit, WST-1 method	A001-3-2	Nanjing Jiancheng Bioengineering Institute, Nanjing, China	Detection range: 0.5-122.0 U/mL; sensitivity: 0.5 U/mL
CAT	Catalase assay kit, ammonium molybdate method	A007-1-1	Nanjing Jiancheng Bioengineering Institute, Nanjing, China	Detection range: 0.2-24.8 U/mL; sensitivity: 0.2 U/mL
MDA	Malondialdehyde assay kit, TBA method	A003-1-2	Nanjing Jiancheng Bioengineering Institute, Nanjing, China	Detection range: 0.5-113.0 nmol/mL; sensitivity: 0.5 nmol/mL
GSH-Px	Glutathione peroxidase assay kit, colorimetric method	A005-1-2	Nanjing Jiancheng Bioengineering Institute, Nanjing, China	Detection range: 20-330 U; sensitivity: 20 U
T-AOC	Total antioxidant capacity assay kit, ABTS method	A015-1-2	Nanjing Jiancheng Bioengineering Institute, Nanjing, China	Detection range: 0.2-55.2 U/mL; sensitivity: 0.2 U/mL
